# The Impact of Histological Variants on Oncological Outcomes After Surgical Resection of a Nonmetastatic Renal Cell Carcinoma with Tumor Thrombus: A Multi-institutional Study

**DOI:** 10.1016/j.euros.2024.02.015

**Published:** 2024-03-08

**Authors:** Raphael Fleury, Théophile Bertail, Karim Bensalah, Jean-Christophe Bernhard, Francois Audenet, Thibaut Waeckel, Bastien Parier, Cécile Champy, Jonathan Olivier, Nicolas Doumerc, Thibault Tricard, Nicolas Branger, Franck Bruyere, Paul Neuville, Louis Surlemont, Jean Alexandre Long, Alexis Fontenil, Maxime Vallee, Morgan Roupret, Romain Boissier, Jean Jacques Patard, Mathieu Durand, Idir Ouzaid, Benjamin Rouget, Xavier Durand, Charlotte Joncour, Olivier Belas, Florie Denise Gomez, Pierre Bigot, Zine-Eddine Khene, members of the French Committee of Urologic Oncology CCAFU

**Affiliations:** a Department of Urology, Centre Hospitalier Universitaire de Rennes, Rennes, France; b LTSI, Inserm U1099, Université de Rennes 1, Rennes, France; c Department of Urology, Centre Hospitalier Universitaire de Bordeaux, Bordeaux, France; e Department of Urology, Hôpital Européen Georges Pompidou, APHP, Paris, France; f Department of Urology, Centre Hospitalier Universitaire de Caen, Caen, France; g Department of Urology, Hôpital Bicêtre, APHP, Paris, France; h Department of Urology, Centre Hospitalier Henri Mondor, APHP, Créteil, France; i Department of urology, Centre Hospitalier Universitaire de Lille, Lille, France; j Department of Urology, Centre Hospitalier Universitaire Rangueil, Toulouse, France; k Department of Urology, Centre Hospitalier Universitaire de Strasbourg, Strasbourg, France; l Department of Urology, Institut Paoli Calmettes de Marseille, Marseille, France; m Department of Urology, Centre Hospitalier Universitaire de Tours, Tours, France; n Department of Urology, Hospices Civils de Lyon, Lyon, France; o Department of Urology, Centre Hospitalier Universitaire de Rouen, Rouen, France; p Department of Urology, Centre Hospitalier Universitaire de Grenoble, Grenoble, France; q Department of Urology, Centre Hospitalier Universitaire de Nîmes, Nîmes, France; r Department of Urology, Centre Hospitalier Universitaire de Poitiers, Poitiers, France; s Department of Urology, Hôpital de la Pitié Salpêtrière, APHP, Paris, France; t Department of Urology, Hôpital de la Conception, APHM, Marseille, France; u Department of Urology, Centre Hospitalier de Mont-de-Marsan, Mont-de-Marsan, France; v Department of Urology, Centre Hospitalier Universitaire de Nice, Nice, France; w Department of Urology, Hôpital Bichat, APHP, Paris, France; x Department of Urology, Centre Hospitalier de Libourne, Libourne, France; y Department of Urology, Hôpital Privé Saint Joseph, Paris, France; z Department of Urology, Centre Hospitalier Universitaire de Reims, Reims, France; aa Department of Urology, Pôle Santé Sud au Mans, Le Mans, France; bb Department of Urology, Hôpital Tenon, APHP, Paris, France; d Department of Urology, Centre Hospitalier Universitaire d’Angers, d’Angers, France

**Keywords:** Kidney cancer, Thrombus, Renal cell carcinoma, Histological variants, Prognosis, Recurrence

## Abstract

**Background:**

There is no definitive evidence of the prognosis impact of histological variants (HVs) in patients who undergo surgical resection of a nonmetastatic renal cell carcinoma (nm-RCC) with venous tumor thrombus (TT).

**Objective:**

To investigate the impact of HVs on the prognosis of patients with nm-RCC with TT after radical surgery.

**Design, setting, and participants:**

Patients who underwent radical nephrectomy with the removal of the venous TT for an nm-RCC were included in a retrospective study.

**Outcome measurements and statistical analysis:**

Three groups were identified: clear cell (ccRCC), papillary (pRCC), and chromophobe (chRCC) RCC. The primary outcome measures (disease-free and overall survival [OS]) were assessed using the Kaplan-Meier method and compared using the log-rank test. Univariate and multivariate Cox proportional hazard models were used to study the impact of HVs on survival.

**Results and limitations:**

A total of 873 patients were included. The histological subtypes were distributed as follows: ccRCC in 780 cases, pRCC in 58 cases, and chRCC in 35 cases. At the time of data analysis, 612 patients were recurrence free and 228 had died. A survival analysis revealed significant differences in both OS and recurrence-free survival across histological subtypes, with the poorest outcomes observed in pRCC patients (*p* < 0.05). In a multivariable analysis, pRCC was independently associated with worse disease-free survival and OS (hazard ratio [HR]: 1.71; *p* = 0.01 and HR: 1.24; *p* = 0.04), while chRCC was associated with more favorable outcomes than ccRCC (HR: 0.05; *p* < 0.001 and HR: 0.02; *p* < 0.001). A limitation of the study is its retrospective nature.

**Conclusions:**

In this multicentric series, HVs appeared to impact the medium-term oncological prognosis of kidney cancer with TT.

**Patient summary:**

This study investigated the differences in oncological outcomes among histological variants (clear cell, papillary, and chromophobe) in a cohort of nonmetastatic renal cell carcinoma patients with venous tumor thrombus extension. We observed that these histological variants within this specific subgroup exhibit distinct outcomes, with papillary renal cell carcinoma being associated with the worst prognosis.

## Take Home Message

Nonmetastatic renal cell carcinoma (RCC) with venous tumor thrombus extension exhibits a unique biological behavior, carrying a 50% risk of progressing to metastatic disease after surgical resection. Given the limited information in the literature concerning the prognostic relevance of histological variants within this group, the aim of our study was to explore the influence of histological variants on prognosis in patients with nonmetastatic RCC and tumor thrombus after undergoing radical surgery. Our findings highlighted a notable disparity across histological subtypes, with papillary RCC being associated with the least favorable survival outcomes.

## 1 Introduction

Renal cell cancer (RCC) with venous involvement (ie, renal vein or caval thrombus) represents approximately 10% of newly diagnosed patients [1], [2]. Without evidence of metastatic disease at diagnosis, surgical removal of the tumor and thrombus is the recommended curative intent treatment [3], [4]. However, this procedure is associated with a significant risk of perioperative morbidity and a high rate of disease recurrence. In fact, the prognosis in this subgroup of patients remains poor, with a high risk of recurrence and overall survival (OS) rates of 40–65% at 5 yr [5].

Among the histological variants of RCC, clear cell RCC (ccRCC) is the commonest histopathological subtype, constituting nearly 80% of cases. It is followed by papillary RCC (pRCC) and chromophobe RCC (chRCC), which account for approximately 15% and 5% of cases, respectively. Each subtype presents a distinct clinical course and varies in treatment response. However, a significant portion of studies including patients with vascular extension focus predominantly on ccRCC alone, or group non–clear cell histologies, such as pRCC and chRCC, into a single category [6], often incorporating mixed cohorts of both metastatic and nonmetastatic diseases [7], [8], [9]. Thus, this approach overlooks the intrinsic association between individual histological variants and the notable differences in patient survival outcomes following radical surgery.

Previous research, including studies by Kim et al [10] and Tilki et al [11], has investigated the impact of histological subtypes, especially pRCC, in patients with RCC and tumor thrombus (TT). These studies, while inclusive of metastatic patients, highlight the variability in survival outcomes based on histological subtypes. Notably, the subset analysis by Tilki et al [11] on nonmetastatic patients identified that the papillary subtype was associated with worse outcomes, pointing to the need for distinct consideration of each subtype.

Owing to the lack of information available in the literature on the prognostic significance of histological variants in the specific population of RCC with TT treated in the modern oncological area, the objective of this study was to investigate the impact of histological variants on prognosis in patients with nonmetastatic RCC with TT following radical surgery.

## 2 Patients and methods

### 2.1 Study design and participants

All patients in this study were prospectively enrolled in the UroCCR multicentric database (UroCCR project [NCT03293563], which is approved by the institutional review board and has obtained the CNIL authorization number DR-2013-206). We conducted a retrospective analysis of all patients who underwent surgical resection of a nonmetastatic RCC with venous TT (renal vein, inferior vena cava, and right atrium), between Jan 1, 2013, and July 31, 2022, at 27 medical centers. Initially, 1077 potentially eligible patients were identified. From these patients, we excluded those with incomplete data about the histological subtype (*n* = 34), with no follow-up data available after surgery (*n* = 103), who received adjuvant or neoadjuvant treatment (*n* = 35), and with a rare type of RCC (*n* = 32).

### 2.2 Tumor characteristics

Histological variants/subtypes were graded according to the 2004 or 2016 World Health Organization classification of kidney tumors, depending on the inclusion date. The tumors were divided into three groups: (1) ccRCC, (2) pRCC, and (3) chRCC. The tumor, node, and metastasis (TNM) stage was recorded according to the 2009 or 2017 TNM classification. The pRCC types 1 and 2 were not distinguished in this cohort.

### 2.3 Follow-up protocol and outcomes

Postoperative follow-up was dependent on the institution and physician, but generally followed national and international guidelines. It usually comprised an outpatient visit at 1 mo postoperatively, then every 6 mo for 3 yr and annually for at least 3 additional years. Follow-up consisted of a disease-specific history assessment, physical examination, and contrast-enhanced computed tomography of the chest, abdomen, and pelvis. The endpoints of interest were disease-free survival (DFS), cancer-specific survival (CSS), and OS. DFS was defined as the time from surgery to disease recurrence (including local and distant recurrences) or death from any cause. For DFS, patients without recurrence and alive were censored at the last follow-up visit. OS was defined as the time from surgery to death from any cause. Deaths attributable to RCC were defined as cancer-specific deaths. CSS was calculated to the date of RCC-associated death. Patients who die from causes unrelated to RCC were considered censored at the time of their death. Patients who are alive were censored at the date of the last contact.

### 2.4 Statistical analysis

Quantitative variables were reported as medians and interquartile ranges (IQRs), and qualitative variables were reported as proportions. The Kruskal-Wallis test was conducted to compare continuous variables. Qualitative variables were compared using the chi-squared and Fisher’s exact tests. A Kaplan-Meier analysis with log-rank tests was performed to estimate the time of recurrence and death from any cause between the groups. Univariate and multivariate Cox proportional hazard models were used to identify independent prognostic factors for DFS, CSS, and OS. Multivariable models included covariates with *p* < 0.2 in a univariate analysis. Various sensitivity analyses were performed. First, the association between histological variants and oncological outcomes stratified by thrombus height (renal vein vs caval thrombosis) was investigated. Second, an analysis repeating cox models were constructed and adjusted for known prognostic factors within each clinical stratum (renal vein vs caval thrombosis). Statistical analyses were performed using Stata 15.1 statistical software (Stata, College Station, TX, USA). All tests were two sided, with a significance level at *p* < 0.05.

## 3 Results

### 3.1 Cohort demographics

A total of 873 RCC patients with venous invasion and no evidence of metastasis at initial presentation were identified. Patient and disease characteristics are shown in Table 1. The histological subtypes were as follows: ccRCC in 780 patients (89.3%), pRCC in 58 patients (6.6%), and chRCC in 35 patients (4%). The pRCC group exhibited a higher rate of lymph node metastasis and more frequently had necrotic areas than the ccRCC and chRCC groups. Patients in the chRCC group tended to be younger (62 yr) and generally in better condition (83% had Eastern Cooperative Oncology Group score 0 and 70% American Society of Anesthesiologists score 1–2). The median (IQR) follow-up of the whole cohort was 28 (10–52) mo. At the time of data analysis, 612 patients were free from recurrence, 228 had died, and 145 deaths were attributable to kidney cancer. The median time to last follow-up among patients still alive with no evidence of events was 31 (IQR 10–56) mo The median follow-up time for patients who died was 25 (8–46) mo.

**Table 1:** Baseline characteristics of patients included in the cohort

	ccRCC (*n* = 780)	pRCC (*n* = 58)	chRCC (*n* = 35)
Age (yr), median (Q1, Q3)	66 (57–73)	69.5 (57–79)	62 (54–68)
Gender, *n* (%)			
Female	233 (29.8)	15 (25.8)	12 (34.2)
BMI (kg/m^2^), median (Q1, Q3)	26.4 (24–30.1)	25.8 (21.8–27.9)	26.25 (23.2–28.4)
Symptomatic presentation, *n* (%)			
Incidental	330 (43)	22 (38.7)	13 (37.1)
Locally symptomatic	314 (40.9)	27 (47.3)	20 (57.1)
Systemically symptomatic	123 (16.1)	8 (14)	2 (5.8)
ASA classification, *n* (%)			
1	125 (17.5)	5 (9.2)	11 (33.4)
2	380 (53.2)	31 (57.4)	19 (57.6)
3	196 (27.4)	14 (26)	3 (9)
4	13 (1.8)	4 (7.4)	0 (0)
ECOG performance status, *n* (%)			
0	490 (73.2)	30 (58.8)	26 (83.8)
≥1	179 (26.8)	21 (41.2)	5 (16.2)
Surgical approach, *n* (%)			
Open	387 (49.6)	43 (74.1)	15 (42.8)
Laparoscopic/robotic	393 (50.3)	15 (25.8)	20 (57.1)
Tumor size (cm), median (Q1, Q3)	7.7 (5.5–10)	9 (8–11)	8 (5.5–11.7)
Nuclear grade, *n* (%)			
1–2	133 (17.4)	4 (7)	NC
3–4	633 (82.6)	53 (93)	NC
Coagulative necrosis, *n* (%)	449 (58)	45 (77.6)	17 (50)
Sarcomatoid features, *n* (%)	173 (22.3)	15 (25.9)	6 (17.6)
Thrombus height, *n* (%)			
Renal vein only	602 (77.1)	34 (58.6)	33 (94.3)
IVC <2 cm	85 (10.9)	12 (20.7)	1 (2.8)
IVC >2 cm	45 (5.7)	8 (13.7)	1 (2.8)
IVC above hepatic veins below diaphragm	33 (4.2)	3 (5.1)	0 (0)
IVC above diaphragm	15 (2)	1 (1.7)	0 (0)
Invasion upper tract urothelial, *n* (%)	171 (22.7)	14 (25)	5 (14.7)
Regional lymph nodes (pN), *n* (%)			
N0	539 (69)	23 (39)	22 (67)
N positive	123 (15)	28 (48)	9 (25)
Nx	118 (15)	7 (12)	4 (11)
Positive surgical margin, *n* (%)	58 (7.4)	8 (13.8)	5 (14.3)

ASA = American Society of Anesthesiologists; BMI = body mass index; ccRCC = clear cell RCC; chRCC = chromophobe RCC; ECOG = Eastern Cooperative Oncology Group; IVC = inferior vena cava; pRCC = papillary RCC; RCC = renal cell carcinoma.

### 3.2 Disease-free survival

**Fig. 1:**
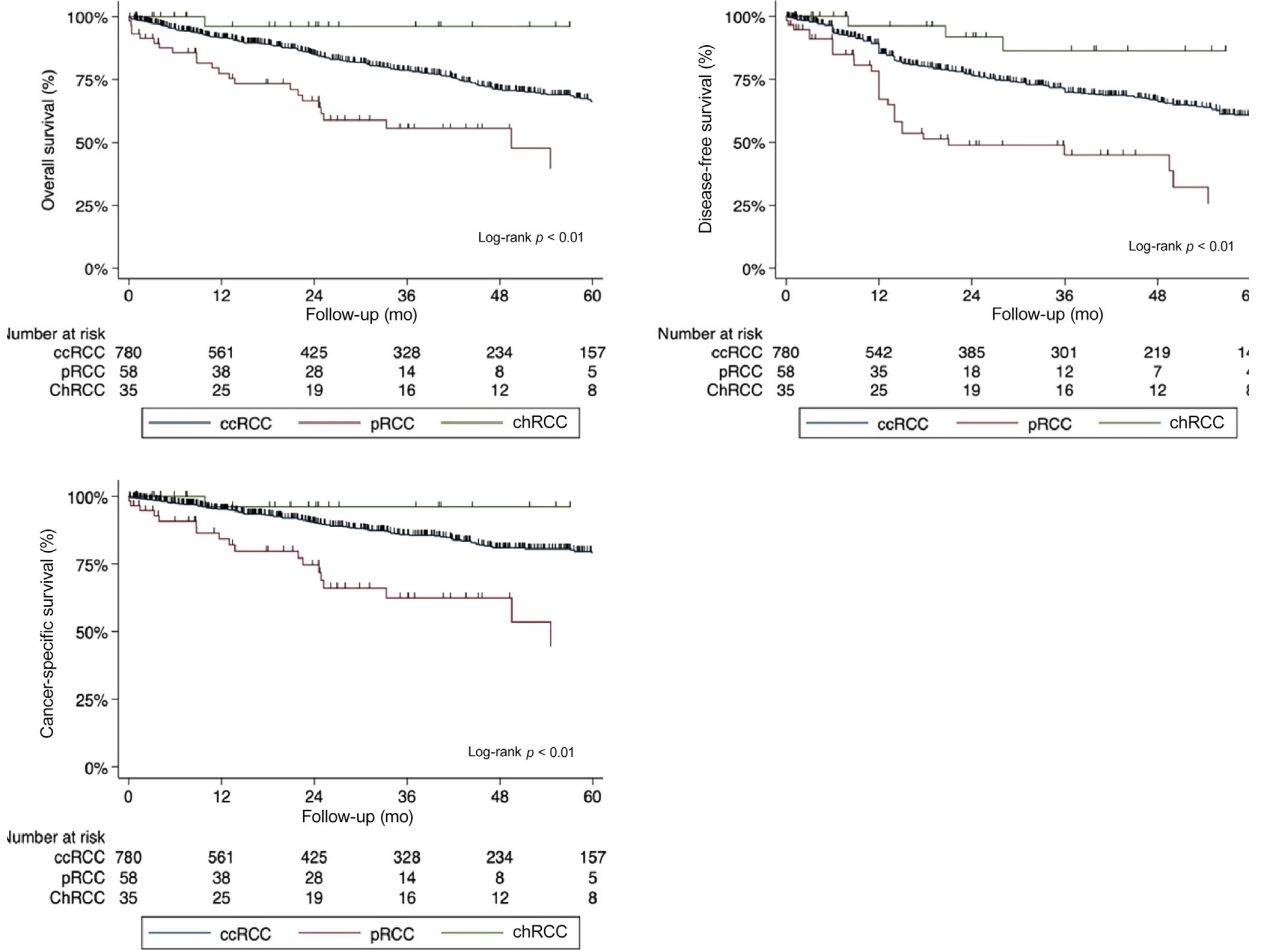
Kaplan-Meier curves showing the estimates of oncological outcomes for patients with nonmetastatic renal cell carcinoma and tumor thrombus treated with surgery for (A) overall survival, (B) disease-free survival, and (C) cancer-specific survival, stratified according to histological variants. ccRCC = clear cell RCC; chRCC = chromophobe RCC; pRCC = papillary RCC; RCC = renal cell carcinoma.

Kaplan-Meier curves for DFS according to histological subtypes are shown in Figure 1. Two-year DFS rates for the cohort stratified according to subtype were 75% (95% confidence interval [CI] 72–79), 49% (95% CI 33–62), and 92% (95% CI 71–97) for ccRCC, pRCC, and chRCC, respectively (*p* = 0.001). On the univariate analysis, features associated with worse DFS were tumor size, nuclear grade, sarcomatoid features, presence of tumor necrosis, thrombus level, presence of nodal disease, positive surgical margins, and histological subtype (all *p* < 0.05). The variables that remained significant on the multivariable analysis included tumor size (*p* < 0.001), presence of nodal disease (*p* = 0.01), and histological variants (*p* = 0.01; Table 2).

**Table 2:** Univariate and multivariable Cox regression analysis assessing the impact of histological variants after surgical resection of a nonmetastatic renal cell carcinoma with tumor thrombus on disease-free survival

	Univariate analysis			Multivariable analysis		
	Hazard ratio	(95% CI)	*p* value	Hazard ratio	(95% CI)	*p* value
Age (increase per 5 yr)	1.02	(0.97–1.07)	0.35			
Tumor size (increase per 10 mm)	2.73	(2.01–3.71)	<0.001	2.46	(1.70–3.55)	<0.001
Necrosis (yes vs no)	1.65	(1.33–2.05)	<0.001	1.26	(0.98–1.71)	0.06
Sarcomatoid features (yes vs no)	1.36	(1.15–1.62)	<0.001	1.18	(0.96–1.49)	0.12
Nuclear grade (1–2 vs 3–4)	2.11	(1.34–3.31)	0.01	1.36	(0.85–2.18)	0.09
Pathological N stage						
pN0	Reference			Reference		
pN1/2	2.11	(1.58–2.81)	<0.001	1.51	(1.01–2.07)	0.01
pNx	1.05	(0.72–1.51)	0.79	0.92	(0.62–1.36)	0.69
Thrombus level (renal vs cava)	1.66	(1.26–2.18)	<0.001	1.13	(0.85–1.52)	0.38
Positive vascular margins (yes vs no)	1.58	(1.01–2.48)	<0.001	1.09	(0.78–2.18)	0.17
Histological variants						
ccRCC	Reference			Reference		
pRCC	2.12	(1.37–3.27)	0.001	1.71	(1.06–2.45)	0.01
chRCC	0.10	(0.01–0.78)	0.028	0.05	(0.01–0.17)	<0.001

ccRCC = clear cell RCC; chRCC = chromophobe RCC; CI = confidence interval; pRCC = papillary RCC; RCC = renal cell carcinoma.

### 3.3 OS and CSS

Survival curves stratified by histological subtype are depicted in Figure 1B. Two-year OS rates for the cohort stratified according to subtype were 82% (95% CI 78–84), 60% (95% CI 46–72), and 97% (95% CI 77–99) for ccRCC, pRCC, and chRCC, respectively (*p* = 0.001). Two-year CSS rates for the cohort stratified according to subtype were 88% (95% CI 86–91), 66% (95% CI 46–78), and 97% (95% CI 76–99) for ccRCC, pRCC, and chRCC, respectively (*p* < 0.001). On the univariate analysis, features associated with worse OS included age, tumor size, nuclear grade, sarcomatoid features, presence of tumor necrosis, thrombus level, and histological subtype of the tumor (all *p* < 0.05). The variables that remained significant on the multivariable analysis included age (*p* = 0.001), tumor size (*p* < 0.001), sarcomatoid features (*p* = 0.03), presence of nodal disease (*p* < 0.001), and histological variants (*p* = 0.05; Table 3).

**Table 3:** Univariate and multivariable Cox regression analysis assessing the impact of histological variants after surgical resection of a nonmetastatic renal cell carcinoma with tumor thrombus on overall survival

	Univariate analysis			Multivariable analysis		
	Hazard ratio	(95% CI)	*p* value	Hazard ratio	(95% CI)	*p* value
Age (increase per 5 yr)	1.13	(1.07–1.21)	<0.001	1.15	(1.08–1.22)	0.001
Tumor size (increase per 10 mm)	2.51	(1.79–3.51)	<0.001	2.28	(1.52–3.41)	<0.001
Necrosis (yes vs no)	1.55	(1.23–1.96)	<0.001	1.51	(0.97–2.07)	0.06
Sarcomatoid features (yes vs no)	1.24	(1.03–1.49)	0.02	1.23	(1.04–1.57)	0.04
Nuclear grade (yes vs no)	1.46	(0.93–2.30)	0.09	1.24	(0.76–2.08)	0.41
Pathologic N stage						
pN0	Reference			Reference		
pN1/2	2.08	(1.53–2.83)	<0.001	1.55	(1.06–2.27)	0.02
pNx	0.87	(0.57–1.32)	0.51	0.94	(0.61–1.48)	0.83
Thrombus level (renal vs cava)	1.64	(1.27–2.12)	<0.001	1.11	(0.81–1.52)	0.58
Positive vascular margins (yes vs no)	1.25	(0.80–1.93)	0.31			
Histological variants						
ccRCC	Reference			Reference		
pRCC	2.31	(1.56–3.43)	<0.001	1.24	(1.08–2.61)	0.04
chRCC	0.35	(0.13–0.95)	0.03	0.02	(0.01–0.12)	<0.001

ccRCC = clear cell RCC; chRCC = chromophobe RCC; CI = confidence interval; pRCC = papillary RCC; RCC = renal cell carcinoma.

### 3.4 Subgroup analysis

**Fig. 2:**
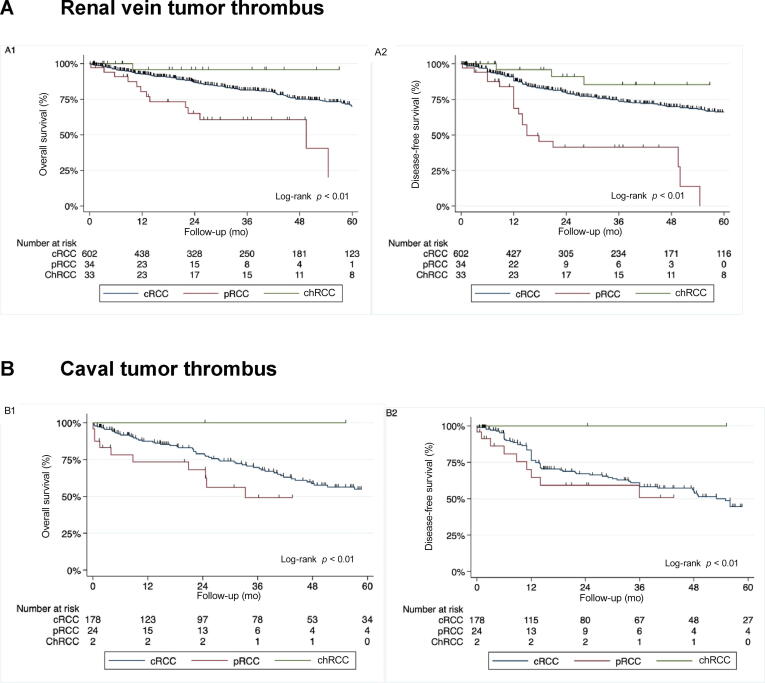
Kaplan-Meier curves showing the estimates of oncological outcomes for patients with nonmetastatic renal cell carcinoma and tumor thrombus treated with surgery, stratified according to histological variants and tumor thrombus level—(A) renal vein and (B) caval thrombosis, for overall survival (A1 and B1) and disease-free survival (A2 and B2). ccRCC = clear cell RCC; chRCC = chromophobe RCC; pRCC = papillary RCC; RCC = renal cell carcinoma.

To test the robustness of our results, we performed various sensitivity analyses. The association between histological variants and oncological outcomes was stratified by thrombus height (renal vein vs caval thrombosis). Similar to the overall cohort, histological variants were associated with both DFS and OS outcomes (Fig. 2A and 2B). Lastly, Cox models were constructed and adjusted for known prognostic factors within each clinical stratum (renal vein vs caval thrombosis). In the multivariable analysis, the histology variants remained significantly associated with DFS and OS (see Supplementary Table 1, Table 2).

## 4 Discussion

Nonmetastatic RCC with venous TT extension has a distinct biological behavior, harboring a 50% risk of progressing to metastatic disease after surgical resection [12]. Consequently, these patients serve as ideal candidates for potential neoadjuvant or adjuvant treatments in clinical studies, making their identification fundamental. However, only few studies have stratified nonmetastatic RCC with venous TT extension according to histological subtype. This oversight could potentially obscure crucial differences in the predictive capabilities of histological subtypes concerning oncological outcomes. We addressed this gap with the current study, which revealed several important observations.

First, most patients with nonmetastatic RCC and TT treated with nephrectomy harbor ccRCC (75.4%), followed by pRCC (16.7%). Only a small minority harbor chRCC (7.9%). Our results are in light with the literatures regarding the epidemiological distribution of RCC subtypes [13]. The overwhelming prevalence of ccRCC underscores the need for therapeutic strategies specifically tailored to this subtype. Additionally, while pRCC and chRCC are less frequent, understanding their unique clinical presentations and molecular characteristics is crucial for optimizing patient care.

Second, we discerned significant differences in patient and cancer characteristics according to histological subtype. In the current analysis, patients with pRCC exhibited a higher rate of lymph node metastasis than those with ccRCC and chRCC. While some previous reports have suggested that pRCC tends to have a higher incidence of lymph node involvement, others have observed a similar trend across all subtypes. Regarding the less common subtype, such as chRCC, the limited number of patients restricts the ability to test the true significance. In our study, a trend was observed with chRCC manifesting as larger tumors, diagnosed at a younger age, and presenting at lower stages and with fewer instances of lymph node invasion compared with the other major subtypes.

Third, analyses specific to histological subtypes revealed important differences in oncological outcomes. Patients with papillary histopathology exhibited significantly worse outcome than those with chromophobe or clear cell subtypes. The histological subtype remained a significant factor associated with both OS and DFS in univariable and multivariable analyses. Although prognostic factors for RCC patients with TT are well established, the influence of histological variants on prognosis continues to be a subject of debate. Margulis et al [14] analyzed the data of 2157 patients comprising those with pRCC (*n* = 245) and ccRCC (*n* = 1912) across all stages. They reported that pRCC patients with venous TT had significantly lower 5-yr CSS than ccRCC patients (35% vs 66%). Tilki et al [11] investigated data from 1774 patients, including those with pRCC (*n* = 151), ccRCC (*n* = 1594), and chRCC (*n* = 29), again encompassing all stages. In their study, the overall 5-yr CSS was 37% for pRCC, 55% for ccRCC, and 59% for chRCC patients. Interestingly, they performed a subgroup analysis restricted to N0M0 patients and found out that histology was significantly associated with CSS in the multivariable analysis.

However, there have been varying conclusions in some research. For example, Terakawa et al [15] and Wagner et al [8] found out that the histological subtype (ccRCC vs others) in TT was a significant prognostic predictor in univariate analyses, but this significance was not maintained in multivariate analyses. Moreover, Kaushik et al [16] found that patients with non-ccRCC and TT did not exhibit a higher rate of disease recurrence or worse survival than those with ccRCC. Conversely, our study demonstrated that histological subtypes with TT were significant factors in multivariate analyses, and the pRCC subtype was associated with worse outcomes, while the chRCC subtype was associated with more favorable outcomes.

Considering our study's findings, particularly the predominance of ccRCC and the notable survival challenges in pRCC, the evolving landscape of immunotherapy in RCC treatment becomes highly relevant. Several recent studies have focused on the impact of immunotherapies in locally advanced high-risk RCC, including those with venous thrombus. For instance, the CheckMate 914 study explored adjuvant nivolumab plus ipilimumab in ccRCC after surgery but found no DFS improvement [17]. In the IMmotion010 trial, adjuvant atezolizumab versus placebo did not delay recurrence in patients with locally advanced ccRCC at a high risk of recurrence after resection [18]. A phase 2 single-center study of 18 patients recently investigated the safety and feasibility of neoadjuvant nivolumab in patients undergoing nephrectomy for localized ccRCC [19]. The results are encouraging, with a safe and feasible treatment without significant surgical delay. The recent KEYNOTE 564 study demonstrated an improvement in recurrence-free survival with pembrolizumab compared with placebo, particularly in the subgroup of patients with locally advanced clear cell renal carcinoma after surgery [20]. These studies underscore the potential of immunotherapy in RCC, particularly in histologies with worse survival outcomes, highlighting the need for further research in this area.

Our study had several important limitations. The major shortcomings were those inherent to the retrospective design. Specifically, the retrospective nature of this evaluation introduces the risk of measurement and ascertainment biases due to nonstandard follow-up. Specifically, the time to diagnosis and progression is largely dependent on when surveillance occurs, and therefore the follow-up protocol employed may influence when the progression event is observed. There were no central pathological review and no standardized interpretation of stage and grade, which may have introduced heterogeneity. Additionally, a significant limitation was the absence of detailed data on the types and locations of, and treatments received for recurrences. This limitation, arising from the incomplete recording of such detailed information across our data sources, hinders a deeper understanding of disease progression and treatment efficacy, marking an essential area for future research. Finally, these data will require external validation in order to confirm the associations reported here.

## 5 Conclusions

This population-based study highlights the critical role of histological subtypes in predicting the oncological outcomes of nonmetastatic patients with RCC and TT, undergoing radical nephrectomy and tumor thrombectomy. Our findings reveal that patients with pRCC and TT have significantly poorer oncological outcomes, while patients with chRCC demonstrate better survival outcomes than those with ccRCC. These insights emphasize the need for tailored therapeutic strategies based on histological subtype to optimize patient outcomes in RCC.

  ***Author contributions*:** Raphael Fleury had full access to all the data in the study and takes responsibility for the integrity of the data and the accuracy of the data analysis.

  *Study concept and design*: Fleury, Khene, Bensalah.

*Acquisition of data*: Fleury, Bertail, Bensalah, Bernhard, Audenet, Waeckel, Parier, Champy, Olivier, Doumerc, Tricard, Branger, Bruyere, Neuville, Surlemont, Long, Fontenil, Vallee, Roupret, Boissier, Patard, Durand, Ouzaid, Rouget, Durand, Joncour, Belas, Gomez, Bigot.

*Analysis and interpretation of data*: Khene, Fleury, Bensalah.

*Drafting of the manuscript*: Fleury, Khene.

*Critical revision of the manuscript for important intellectual content*: All authors.

*Statistical analysis*: Fleury, Khene, Bensalah.

*Obtaining funding*: None.

*Administrative, technical, or material support*: None.

*Supervision*: Khene, Bensalah.

*Other*: None.

  ***Financial disclosures:*** Raphael Fleury certifies that all conflicts of interest, including specific financial interests and relationships and affiliations relevant to the subject matter or materials discussed in the manuscript (eg, employment/affiliation, grants or funding, consultancies, honoraria, stock ownership or options, expert testimony, royalties, or patents filed, received, or pending), are the following: None.

  ***Funding/Support and role of the sponsor*:** All patients in this study (UroCCR n121) were enrolled in the UroCCR multicentric database (ClinicalTrials.gov: NCT03293563/CNIL agreement DR-2013-206, CPP: DC 2012/108). All patients received oral and written information about the objectives and methodology of the UroCCR project and written consent was obtained.

  ***Acknowledgments*:** Z-E Khene would like to thank the French Association of Urology for their support and assistance (AFU/2022).
