# Supplementary material for: The Impact of Histological Variants on Oncological Outcomes After Surgical Resection of a Nonmetastatic Renal Cell Carcinoma with Tumor Thrombus: A Multi-institutional Study
Source: Eur Urol Open Sci. 2024 Mar 8;62:123–30. doi: 10.1016/j.euros.2024.02.015 (PMC10940768; doi:10.1016/j.euros.2024.02.015)
Supplement: Supplementary data 1 [file mmc1.docx]

**Supplementary Table 1.** *Multivariable Cox Regression Analysis Assessing the Impact of histological variants after surgical resection of a Nonmetastatic Renal Cell Carcinoma with Tumor Thrombus on overall survival (OS) and disease-free survival (DFS): renal vein thrombus subgroup.*

|  | Multivariable analysis | | | Multivariable analysis | | |
| --- | --- | --- | --- | --- | --- | --- |
|  | DFS | | | OS | | |
|  | Hazard ratio | (95% CI) | P | Hazard ratio | (95% CI) | P |
| Age | 1.00 | (0.99-1.02) | 0.27 | 1.03 | (1.01-1.04) | <0.001 |
| Tumour size | 1.09 | (1.04-1.15) | <0.01 | 1.09 | (1.03-1.15) | 0.001 |
| Necrosis | 1.34 | (0.94-1.83) | 0.11 | 1.47 | (1.04-2.07) | 0.02 |
| Sarcomatoid features | 1.36 | (1.08-1.75) | 0.01 | 1.26 | (0.96-1.62) | 0.08 |
| Nuclear grade | 1.75 | (1.02-2.97) | 0.04 | 1.35 | (0.79-2.29) | 0.26 |
| Pathologic N-stage |  |  |  |  |  |  |
| pN0 | Ref |  |  | Ref |  |  |
| pN1/2 | 1.34 | (1.02-2.01) | 0.04 | 1.83 | (1.08-2.09) | 0.03 |
| pNx | 0.91 | (0.59-1.53) | 0.85 | 0.91 | (0.49-1.42) | 0.51 |
| Histological variants |  | | |  | | |
| cRCC | Ref |  |  | Ref |  |  |
| pRCC | 2.50 | (1.48-4.23) | 0.001 | 1.66 | (1.01-3.12) | 0.03 |
| Ch RCC | 0.006 | (0.001-0.09) | <0.001 | 0.01 | (0.001-0.11) | <0.001 |

**Supplementary Table 2.** *Multivariable Cox Regression Analysis Assessing the Impact of histological variants after surgical resection of a Nonmetastatic Renal Cell Carcinoma with Tumor Thrombus on overall survival (OS) and disease-free survival (DFS): Caval tumor thrombus subgroup*

|  | Multivariable analysis | | | Multivariable analysis | | |
| --- | --- | --- | --- | --- | --- | --- |
|  | DFS | | | OS | | |
|  | Hazard ratio | (95% CI) | P | Hazard ratio | (95% CI) | P |
| Age | 1.006 | (0.98-1.02) | 0.48 | 1.02 | (1.01-1.04) | 0.02 |
| Tumour size | 1.08 | (1.02-1.14) | 0.008 | 1.06 | (1.006-1.13) | 0.05 |
| Necrosis | 1.11 | (0.65-1.90) | 0.69 | 0.98 | (0.57-1.68) | 0.94 |
| Sarcomatoid features | 1.05 | (0.73-1.51) | 0.78 | 1.08 | (0.74-1.58) | 0.66 |
| Nuclear grade | 0.97 | (0.39-2.40) | 0.94 | 1.61 | (0.55-4.71) | 0.36 |
| Pathologic N-stage |  |  |  |  |  |  |
| pN0 | Ref |  |  | Ref |  |  |
| pN1/2 | 1.79 | (1.05-3.07) | 0.03 | 1.81 | (1.03-3.15) | 0.03 |
| pNx | 0.96 | (0.47-1.96) | 0.92 | 0.78 | (0.37-1.65) | 0.53 |
| Histological variants |  | | |  | | |
| cRCC | Ref |  |  | Ref |  |  |
| pRCC | 1.11 | (1-2.05) | 0.03 | 1.21 | (1.09-2.41) | 0.04 |
| * The ChRCC group was not included in the analysis due to small numbers (n=2). | | | | | | |
